# Humanized TLR4/MD-2 Mice Reveal LPS Recognition Differentially Impacts Susceptibility to *Yersinia pestis* and *Salmonella enterica*

**DOI:** 10.1371/journal.ppat.1002963

**Published:** 2012-10-11

**Authors:** Adeline M. Hajjar, Robert K. Ernst, Edgardo S. Fortuno, Alicia S. Brasfield, Cathy S. Yam, Lindsay A. Newlon, Tobias R. Kollmann, Samuel I. Miller, Christopher B. Wilson

**Affiliations:** 1 Department of Comparative Medicine, University of Washington, Seattle, Washington, United States of America; 2 Department of Microbial Pathogenesis, University of Maryland, Baltimore, Maryland, United States of America; 3 Division of Infectious and Immunological Diseases, University of British Columbia, Vancouver, British Columbia, Canada; 4 Department of Immunology, University of Washington, Seattle, Washington, United States of America; 5 Departments of Medicine, Genome Sciences, and Microbiology, University of Washington, Seattle, Washington, United States of America; Portland VA Medical Center/Oregon Health and Science University, United States of America

## Abstract

Although lipopolysaccharide (LPS) stimulation through the Toll-like receptor (TLR)-4/MD-2 receptor complex activates host defense against Gram-negative bacterial pathogens, how species-specific differences in LPS recognition impact host defense remains undefined. Herein, we establish how temperature dependent shifts in the lipid A of *Yersinia pestis* LPS that differentially impact recognition by mouse versus human TLR4/MD-2 dictate infection susceptibility. When grown at 37°C, *Y. pestis* LPS is hypo-acylated and less stimulatory to human compared with murine TLR4/MD-2. By contrast, when grown at reduced temperatures, *Y. pestis* LPS is more acylated, and stimulates cells equally via human and mouse TLR4/MD-2. To investigate how these temperature dependent shifts in LPS impact infection susceptibility, transgenic mice expressing human rather than mouse TLR4/MD-2 were generated. We found the increased susceptibility to *Y. pestis* for “humanized” TLR4/MD-2 mice directly paralleled blunted inflammatory cytokine production in response to stimulation with purified LPS. By contrast, for other Gram-negative pathogens with highly acylated lipid A including *Salmonella enterica* or *Escherichia coli*, infection susceptibility and the response after stimulation with LPS were indistinguishable between mice expressing human or mouse TLR4/MD-2. Thus, *Y. pestis* exploits temperature-dependent shifts in LPS acylation to selectively evade recognition by human TLR4/MD-2 uncovered with “humanized” TLR4/MD-2 transgenic mice.

## Author Summary

The outer leaflet of the outer membrane of Gram-negative bacteria is mainly composed of lipopolysaccharide (LPS, endotoxin). The structure of the bioactive component of LPS, lipid A, varies between bacteria and even within the same species grown under different environmental conditions. *Yersinia pestis* has been associated with highly lethal bubonic plagues of the past. It alters the structure of its LPS based on temperature. When grown at ambient temperatures comparable to fleas in temperate climates, the LPS is mainly hexa-acylated. However, upon growth at 37°C, the mammalian host temperature, *Y. pestis* switches to synthesize a hypo-acylated LPS that is less stimulatory to the human compared with murine LPS receptor complex composed of Toll-like receptor (TLR) 4 and MD-2. To test whether the change in LPS structure associated with replication at mammalian temperature promotes *Y. pestis* virulence by evading recognition by the human receptor complex, we generated “humanized” mice that express human rather than mouse TLR4 and MD-2. We find that these mice are indeed more sensitive to *Y. pestis* infection than WT mice supporting the notion that evasion of recognition by TLR4/MD-2 promotes *Y. pestis* virulence in humans.

## Introduction

The activation of host defense against Gram-negative bacterial pathogens is initiated after recognition of the bioactive component of lipopolysaccharide (LPS), lipid A, through Toll-like receptor (TLR) 4 and its coreceptor MD-2 [1]–[4]. Variations in lipid A structure exist among diverse bacterial species, and interestingly can also occur within the same bacteria after growth in different environmental conditions [5]–[7]. In turn, variations in lipid A have been proposed to influence species-specific innate immune recognition. For example, enteric pathogens such as *Escherichia coli* and *Salmonella enterica* express lipid A that is mainly hexa-acylated and highly stimulatory to both mouse and human TLR4/MD-2. However, hypoacylated LPS (with penta- or tetra-acylated lipid A) is poorly recognized by the human compared with mouse receptor complex resulting in a reduced inflammatory response [6], [8], [9]. These discordant responses suggest experimental infection in conventional mice may not accurately model the complex immune evasion strategies related to lipid A structural modifications employed by some Gram-negative bacterial pathogens that selectively cause human infection.

*Yersinia pestis* evolved from the enterically transmitted pathogen *Y. pseudotuberculosis* approximately 1,500–20,000 years ago, acquiring the ability to replicate in the flea intestine that facilitates transmission through this intermediate vector [10]. When grown at ambient temperatures that mimic fleas in temperate climates, the lipid A is mainly hexa-acylated. However, upon growth at higher temperature (37°C) representative of mammalian hosts, *Y. pestis* instead synthesizes a predominantly tetra-acylated lipid A structure that selectively stimulates rodent compared with the human LPS receptor complex TLR4/MD-2 [11]–[13]. The potential importance of these shifts in *Y. pestis* lipid A as an immune evasion tactic is supported by the attenuation of strains with forced expression of a hexa-acylated structure due to heterologous expression of *E. coli lpxL* lauryl acyltransferase enzyme after infection in WT, but not TLR4-deficient, mice [14]. Reciprocally, administration of TLR4 agonists augments host defense against *Y. pestis* further highlighting the potential importance shifts in TLR4 recognition play in infection susceptibility [15]. Importantly however, these findings using TLR4-deficient or control mice did not address how the discordance in LPS recognition by human compared with mouse TLR4/MD-2 impacts host defense after infection in humans. To bypass these limitations, “humanized” mice that express human TLR4/MD-2 instead of each respective molecule in mice were generated. Using these newly developed transgenic mouse tools, the impacts whereby blunted recognition of hypoacylated LPS by human TLR4/MD-2 dictates susceptibility to pathogens like *Y. pestis* that shift lipid A structure *in vivo* are uncovered.

## Results

### Reduced potency of *Y. pestis* grown at 37°C for human TLR4/MD-2

**Figure 1:**
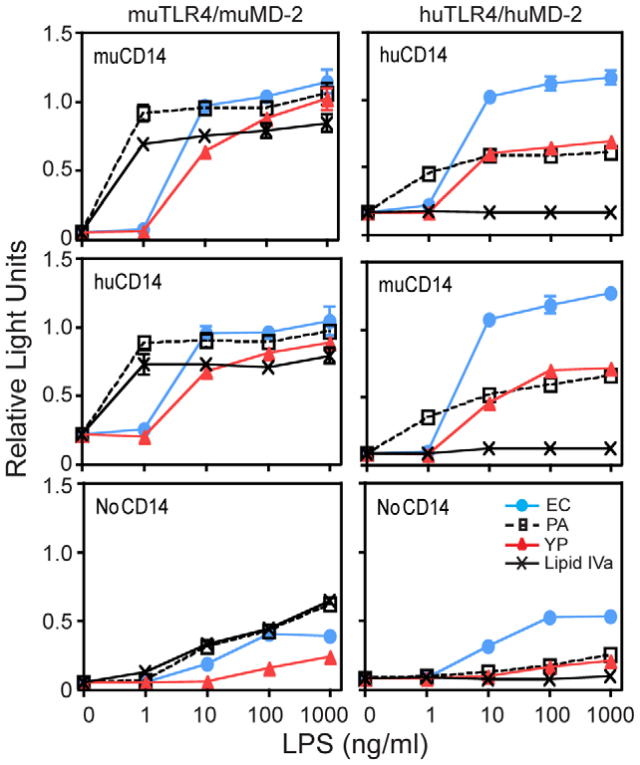
Decreased responsiveness to LPS of *Y. pestis* grown at 37°C by huTLR4/MD-2 *in vitro*. HEK-293 cells expressing the indicated proteins were stimulated for 4 hr with increasing concentrations of *E. coli* (EC, blue), *P. aeruginosa* (PA), or *Y. pestis* (YP, red, grown at 37°C) LPS or Lipid IVa. Firefly luciferase was induced by NF-kB activation (ELAM promoter) and corrected by the constitutively active ß-actin driven Renilla luciferase. Relative Light Units plotted are the direct Firefly/Renilla values. Shown is the average (+/−SD) of triplicate wells from one representative experiment of 2. See Table S1 for statistical analysis.

We first investigated the differential recognition of LPS from *Y. pestis* and other Gram-negative bacteria in HEK-293 cells transiently transfected with either mouse or human *TLR4/MD-2*, with or without mouse or human *CD14* (Fig. 1, statistical analysis shown in Table S1). Cells transfected with human (hu) *TLR4*, hu*MD-2*, and hu*CD14* induced the NF-kB-driven luciferase reporter robustly in response to hexa-acylated *E. coli* (EC) LPS. By contrast, the response to tetra-acylated *Y. pestis* (YP) LPS was sharply reduced and similar to the blunted response to penta-acylated *Pseudomonas aeruginosa* (PA) LPS that we have previously described [16] (6.8-fold for EC vs. 4.0- and 3.6-fold induction for YP and PA over unstimulated levels at 1000 ng/ml of each LPS; see Fig. S1 for lipid A structures). Importantly, the reduced potency of YP LPS was specific for recognition by human TLR4/MD-2/CD14 because all 3 LPS preparations stimulated cells transfected with murine (mu) *TLR4/MD-2/CD14* to a similar extent (20-, 19- and 18-fold induction over unstimulated levels at 1000 ng/ml of EC, PA, and YP LPS). As predicted [17], cells transfected with mu*TLR4*/mu*MD-2*/mu*CD14* but not hu*TLR4*/hu*MD-2*/hu*CD14* responded to synthetic tetra-acylated lipid A (Lipid IVa, 15- vs 1-fold induction at 1000 ng/ml). Additional transfection studies demonstrated that the reduced responsiveness for hypoacylated lipid A is directly attributable to species-specific differences between human and mouse TLR4 and MD-2, while the species source of CD14 did not contribute in significant fashion (Fig. 1) as previously shown [18]. Together, these *in vitro* results illustrate species-specific variations in cell stimulation by hypoacylated LPS, and suggest that these lipid A modifications may contribute to selective immune evasion in humans for specific Gram-negative bacterial pathogens.

### Generation of “humanized” TLR4/MD-2 mice

**Figure 2:**
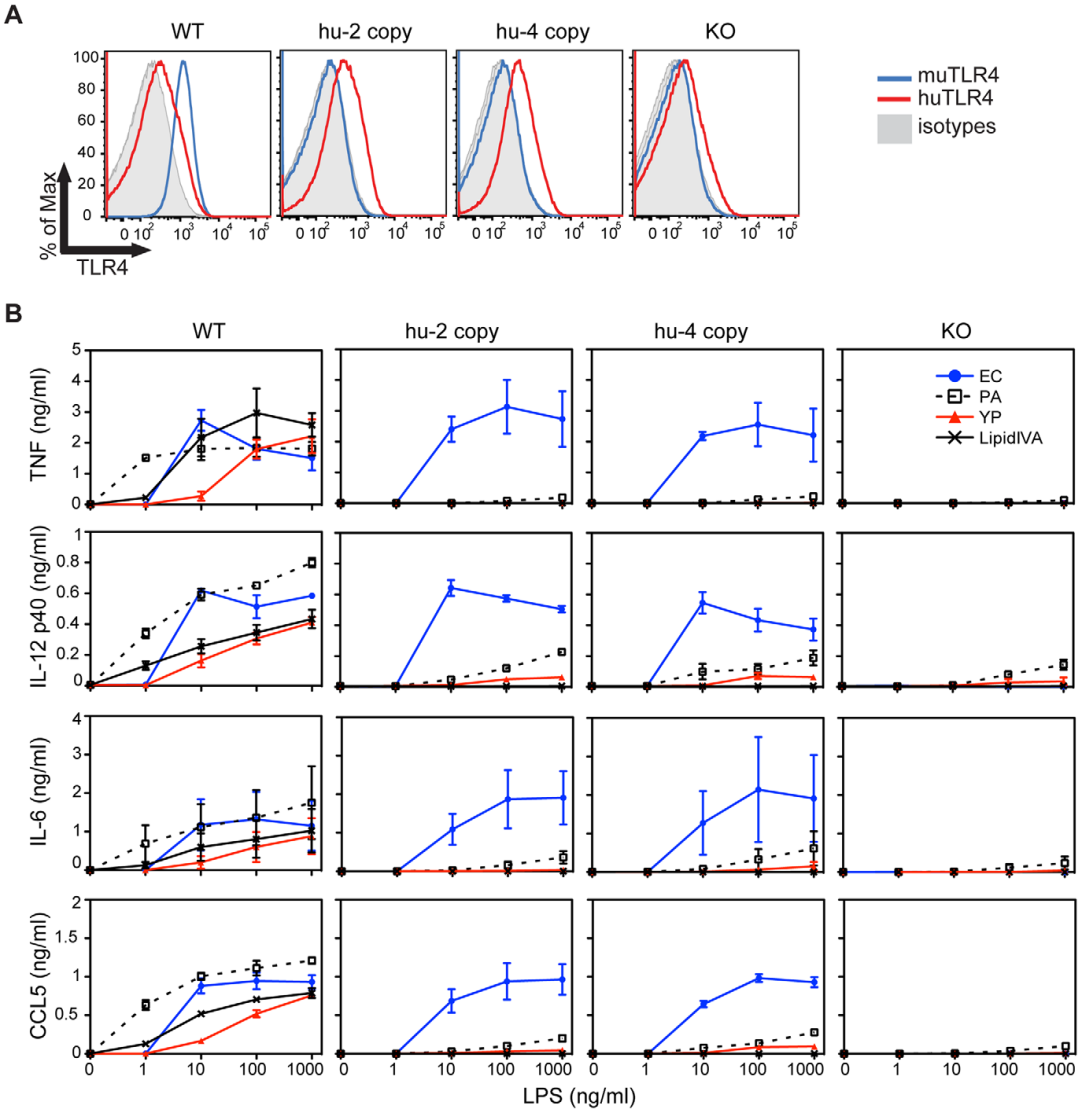
Decreased responsiveness to hypoacylated LPS by BMDM from humanized TLR4/MD-2 mice. (A) BMDM (CD11b+ F4/80+ cells) were stained for surface TLR4 expression at the time of harvest, prior to stimulation. Blue histograms depict anti-mouse TLR4/MD-2 staining, red histograms anti-human TLR4/MD-2 and filled grey histograms isotype controls. Shown is one representative experiment of 2. (B) BMDM from the indicated mice were stimulated with increasing concentrations of LPS for 4 hr and cytokines/chemokines were measured in the supernatant by Luminex. Shown are the mean and range of 2 independent experiments (one well per stimulation per experiment).

To further investigate the hypothesis that reduced recognition of hypoacylated lipid A contributes to *Y. pestis* immune evasion in humans, a genomic bacterial artificial chromosome (BAC) transgenic approach was used to direct tissue-specific expression of human TLR4 and MD-2. Mice transgenic for human BACs encoding TLR4 and MD-2 were intercrossed with mice containing targeted deletions of both mouse *TLR4* and *MD-2* thereby replacing the mouse receptors with the human receptors. Two independent hu*TLR4* BAC+ lines were generated, with either 2 or 4 copies of the transgene, and expression of huTLR4 was confirmed with a trend towards higher message levels in the 4-copy line (Fig. S2). However, using a similar approach, only a single hu*MD-2* BAC+ line was successfully generated following 10 independent injections, and this integration event appears to occur on the Y-chromosome as 100% of males, and 0% of female mice, were found to encode huMD-2. Therefore, only male transgenic and control mice were used in future studies. Intercrossing these two transgenic mouse strains with other mice containing targeted defects in mouse *TLR4* and *MD-2* leads to “humanized” TLR4/MD-2 mice that exclusively express human TLR4 and MD-2 and lack endogenous murine TLR4 and MD-2. Surface expression of huTLR4/MD-2 was detected in bone-marrow derived macrophages from these mice (Fig. 2A), and cytokine production by these cells paralleled the differences in responsiveness in transfected HEK293 cells (Fig. 2B).

### Humanized TLR4/MD-2 mice recapitulate the differential recognition of LPS from *Y. pestis* and *E. coli*

**Figure 3:**
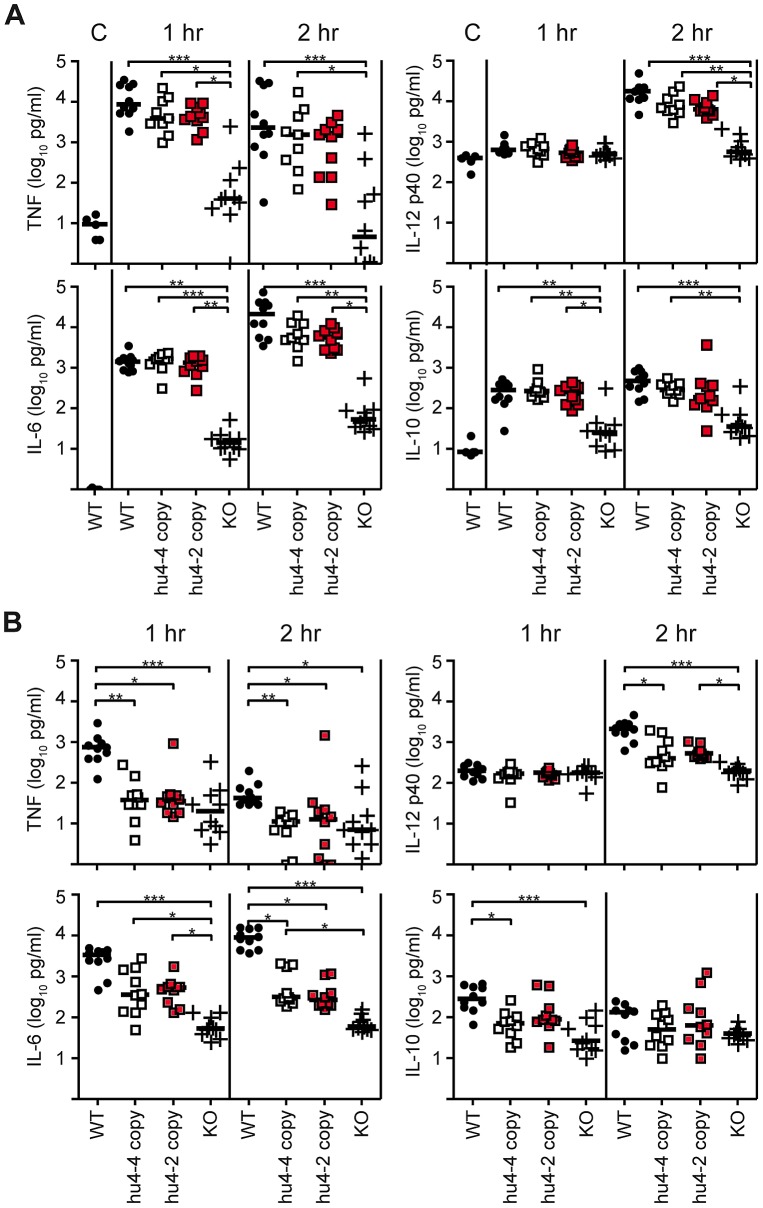
Complete reconstitution of EC but only partial reconstitution of *Y. pestis* LPS responsiveness *in vivo* in humanized TLR4/MD-2 mice. WT, 2- and 4-copy humanized, and KO mice were injected intraperitoneally with 100 µg EC LPS (A) or YP LPS (B). Serum was collected from the same animals at 1 and 2 hr post injection. Baseline levels in control (C) unmanipulated WT mice were also measured. Each symbol represents one animal. Shown are the pooled results of 2 separate experiments (N = 9–10 total per group); line represents median. Data were analyzed using 1-way ANOVA followed by Dunn's post-test for multiple pairwise comparisons. Significant differences are shown. *** *P*<0.001, **P<0.01, *P<0.05.

To determine if the *in vivo* response to hexa-acylated EC LPS, which is strongly agonistic for human as well as mouse receptors, was restored in KO mice transgenic for human *TLR4/MD-2*, we measured the induction of pro-inflammatory cytokines by EC LPS. The serum levels of TNF, IL-6 and IL-12/IL-23 p40 each increased markedly, and IL-10 increased modestly, in humanized TLR4/MD-2 transgenic mice, and the levels of these cytokines were not significantly different between mice with either 2- or 4-copies of the hu*TLR4* BAC transgene compared with control mice expressing endogenous mouse TLR4/MD-2 (Fig. 3A). As expected, only background levels were found in *TLR4/MD-2* KO mice illustrating the importance of TLR4/MD-2 in this response. By contrast, after stimulation with *Y. pestis* LPS (after growth at 37°C), the induction of IL-6 and IL-12/IL-23 p40 was sharply reduced in humanized TLR4/MD-2 mice compared with WT mice (Fig. 3B). Furthermore, the induction of TNF and IL-10 were completely extinguished in humanized TLR4/MD-2 mice and found at levels similar to *TLR4/MD-2* KO mice (Fig. 3B). Together, these results demonstrate that while humanized TLR4/MD-2 mice have the capacity to respond vigorously and to the same extent as WT mice after stimulation with hexa-acylated EC LPS, their response is blunted when *Y. pestis* LPS is used for stimulation.

### Cells from mice humanized for TLR4/MD-2 respond poorly to LPS from *Y. pestis* grown at 37°C

**Figure 4:**
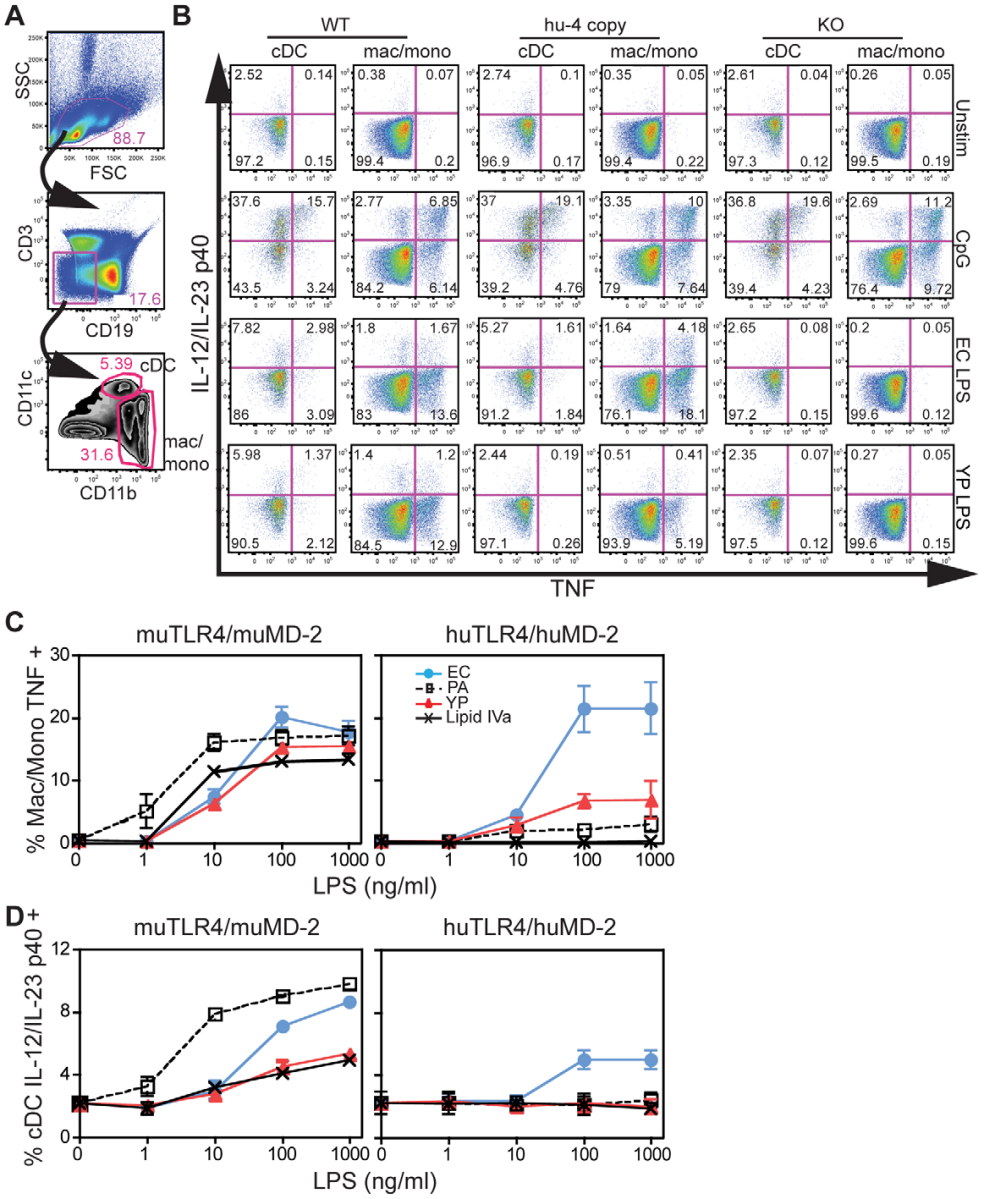
Decreased responsiveness to LPS of *Y. pestis* grown at 37°C by splenocytes from 4-copy humanized TLR4/MD-2 mice. (A) Gating strategy to identify mac/mono and cDC. CD19/CD3 double negative cells from the live gate were further gated on CD11c high/CD11b+cells (cDC) or CD11c low to negative/CD11b+cells (mac/mono). (B) Splenocytes were either left unstimulated (unstim, medium alone), or stimulated with CpG (ODN1826, 10 µg/ml) or EC or YP LPS (1 µg/ml) for 4 hr in the presence of BFA. Numbers in quadrants indicate the % of cells in the panel that are found in that quadrant. Therefore TNF+ cells are the sum of the right 2 quadrants and IL-12/IL-23 p40+ cells the sum of the top 2 quadrants. (C) % of macrophages/monocytes producing TNF and (D), % cDC producing IL-12/IL-23 p40 in response to each stimulation. Shown are the mean values (+/−SD) of 3 separate experiments. See Table S2 for statistical analyses.

We next developed a multi-color flow-based assay, as described for human whole blood and PBMC [19], [20], to enable simultaneous examination of cytokine production in distinct primary cell populations (Fig. 4A). Analysis of the relative composition of various cellular subsets in the spleen revealed no differences between WT and humanized TLR4/MD-2 mice (Fig. S3). Surface expression of huTLR4/MD-2 was detected only in macrophages, although muTLR4/MD-2 was also detected in B-cells (Fig. S4). Using this gating strategy, we found that mouse macrophages/monocytes preferentially produced TNF whereas conventional dendritic cells (cDC) produced IL-12/IL-23 p40 in response to stimulation with either LPS or CpG (Fig. 4B). TNF production by mac/mono in response to the LPS preparations from the various species paralleled the differences in responsiveness in transfected HEK293 cells (Fig. 4C, see Table S2 for statistical analysis). Primary mac/mono from humanized TLR4/MD-2 mice responded preferentially to EC LPS, whereas cells from WT mice responded robustly to all four preparations, similar to what we observed with bone-marrow derived macrophages (Fig. 2B). cDC from WT mice were less responsive (i.e. a fewer percentage of the cells responded) to LPS than mac/mono, and showed greater discrimination of hypo-acylated molecules (as shown previously [21]), possibly due to the lack of membrane CD14 expression in cDC compared to mac/mono (Fig. 4B and 4D). Moreover, cDC from humanized TLR4/MD-2 mice responded only to EC LPS. Taken together, these data demonstrate that cells from humanized TLR4/MD-2 mice responded poorly to hypoacylated LPS preparations, as do cells from humans.

### Selective susceptibility to *Y. pestis* in humanized TLR4/MD-2 mice

**Figure 5:**
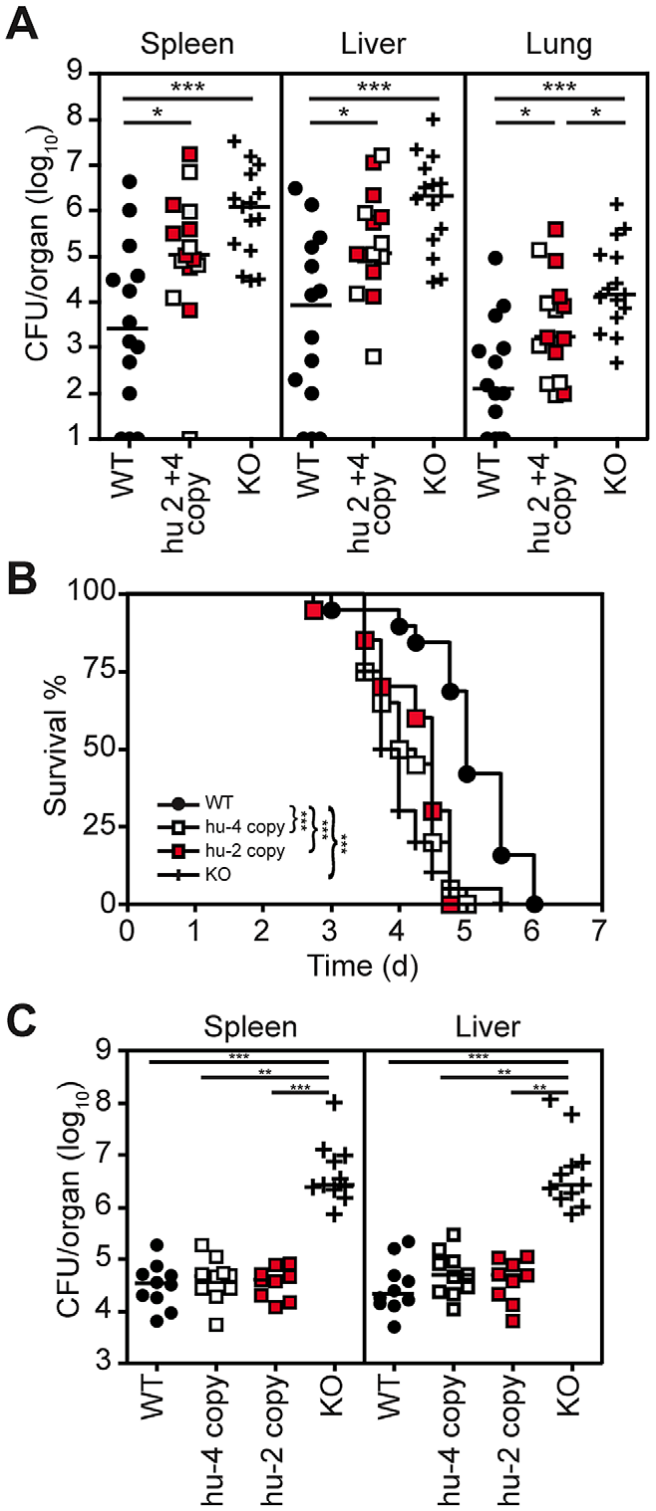
Increased susceptibility to *Y. pestis* infection but effective control of *S.* Typhimurium infection in humanized TLR4/MD-2 mice. (A) WT, KO, and humanized mice were infected with ∼274 CFU CO92 subcutaneously and CFU in the spleen, liver, and lungs were measured 72 hr later. Shown are the combined results of 5 separate experiments (N = 14 WT, 7 4-copy [white-filled squares] and 8 2-copy [red-filled squares] humanized TLR4/MD-2, and 15 KO mice). Line represents median and data were analyzed using the Mann-Whitney test performing pairwise comparisons. Significant differences are shown. *** *P*<0.001, *P<0.05. (B) WT, KO, 4-copy and 2-copy humanized mice were infected with ∼200 CFU CO92 subcutaneously. The log-rank test was used to compare the survival curves. WT mice died significantly later than 4-copy or 2-copy humanized or KO mice (*P*<0.0001). Combined data from 2 separate experiments are shown (N = 19–20 total per group). (C) WT, KO, 4-copy and 2-copy humanized mice were infected with ∼1,700 CFU SL1344 and CFU in the liver and spleen were measured 48 hr later. Shown are the combined results from 2 separate experiments (N = 9–10 total per group); line represents median. Data were analyzed using 1-way ANOVA followed by Dunn's post-test for multiple pairwise comparisons. Significant differences are shown. *** *P*<0.001, **P<0.01.

To investigate the impact of human LPS recognition specificity on host defense against *Y. pestis*, the relative susceptibility after infection was compared in WT, KO, and humanized TLR4/MD-2 mice. To recapitulate the natural route of infection for bubonic plague, mice were injected subcutaneously with ∼200 CFU of virulent *Y. pestis* (strain CO92) grown at 37°C and CFU and survival were monitored thereafter. We found significantly higher counts in the humanized TLR4/MD-2 and KO mice compared to WT 72 hr after infection despite the large spread in each group (median CFU for spleen, liver, and lung of 1.06×10^5^, 1.14×10^5^, and 1.67×10^3^ in humanized and 1.19×10^6^, 2.08×10^6^, and 1.41×10^4^ in KO vs. 2.48×10^3^, 8.12×10^3^, and 1.3×10^2^ in WT, Fig. 5A). The CFU in the lungs of humanized TLR4/MD-2 mice were also significantly reduced compared to KO. When we examined survival, we found that humanized TLR4/MD-2 mice succumbed to infection more rapidly compared with WT control mice, and the time to death in humanized TLR4/MD-2 mice was similar to TLR4/MD-2 KO mice (Fig. 5B). Specifically, the median survival for WT mice was 5 days compared to 3.9 days for KO mice and 4.1 days for the 4-copy and 4.5 days for the 2-copy humanized mice, indicating that reduced recognition of tetra-acylated lipid A by the human receptor complex resulted in more rapid disease progression *in vivo*. We saw similar results when mice were infected with hexa-acylated *Y. pestis* grown at 26°C (Fig. S5), indicating that the inoculum itself was probably not triggering TLR4 but that the rapid *in vivo* replication, at 37°C, was most likely providing the tetra-acylated ligand.

In contrast to *Y. pestis*, *S.* Typhimurium hexa-acylated LPS is highly stimulatory to both mouse and human TLR4/MD-2. Furthermore, control of infection is dependent on TLR4 [22]–[25]. Therefore, to determine the relative contribution of TLR4/MD-2 in control of a bacterium with equally stimulatory LPS, WT, KO and humanized TLR4/MD-2 mice were challenged intraperitoneally with *S.* Typhimurium. Spleen and liver bacterial counts in humanized mice were similar to those in WT mice (median of 4.7×10^4^ and 4×10^4^ vs. 3.45×10^4^ CFU for spleen in 4-copy and 2-copy humanized vs. WT mice, and 4.9×10^4^ and 4.86×10^4^ vs. 2.17×10^4^ CFU for liver) and significantly lower than those in KO mice (2.59×10^6^ in the spleen and 2.64×10^6^ in the liver, Fig. 5C). These data demonstrate that expression of human TLR4 and MD-2 from genomic BACs effectively reconstitutes the mouse KO and results in a functionally coordinated response to infection with bacteria with highly stimulatory LPS.

## Discussion

Our results indicate that alteration of the specificity of the LPS recognition pathway is sufficient to affect the outcome of infection with *Y. pestis* but not *S.* Typhimurium. More specifically, our findings demonstrate that the switch from hexa-acylated to tetra-acylated lipid A may contribute to the virulence of *Y. pestis* in humans and that the impact of this switch on virulence is not accurately reflected in conventional mouse models. Similarly, conventional mice may not accurately model the virulence of other pathogens whose lipid A structures are differentially recognized by human and mouse LPS receptor complexes, including *P. aeruginosa* [16], *Bordetella pertussis* [26], *Leptospira interrogans* [27] and *Neisseria meningitidis* [28]. Unfortunately, the novel humanized mouse model described here is limited to male mice and therefore will not allow studies on the increased susceptibility to *Y. pestis* described for males relative to females [29], [30]. It should also be noted that the human TLR4-mediated responses that we evaluated were mostly MyD88-dependent responses although we measured the TRIF-dependent chemokine CCL5 [31] *in vitro* (Fig. 2B). Monophosphoryl lipid A, a licensed human adjuvant, has been shown to preferentially trigger the TRIF pathway downstream of TLR4 [32], perhaps promoting its enhanced adjuvant effect compared to LPS. Furthermore, novel synthetic TLR4 adjuvants preferentially trigger the TRIF pathway [33]. It will be interesting to test these compounds in the humanized TLR4/MD-2 mice.

We were astonished to measure a significant response to LPS from *Y. pestis* grown at 37°C in cells transfected with the human receptor complex as well as in cells from the humanized mice. The *Y. pestis* tetra-acylated lipid A structure is essentially that of Lipid IVa, a well-characterized antagonist of human TLR4/MD-2 [17]. The increased response to YP LPS compared to Lipid IVa may be due to the presence of pyrophosphate and aminoarabinose [34] or core saccharides in the purified LPS that would be absent in synthetic Lipid IVa, especially considering the crucial role that phosphates play in binding and signaling via TLR4/MD-2 [35]. Alternatively, the presence of additional lipid A species such as penta-acylated molecules (shown in [12]), or even differences in solubility between Lipid IVa and YP LPS, may also contribute to the increased signal.

Individuals with common polymorphisms in the extracellular portion of TLR4 are less responsive to EC LPS (reviewed in [6]), with evidence that they may also be less responsive to hypoacylated LPS [36]. It is possible that specific polymorphisms resulting in reduced cytokine responses and increased susceptibility to Gram-negative infections may have been selected for during the large outbreaks of plague in the Middle Ages [37]. However, it remains to be determined whether these human variants affect outcome following *Y. pestis* infection and could have contributed to this selection.

Surprisingly, the more virulent *Y. pestis* recently evolved from *Y. pseudotuberculosis* through the gain of a limited amount of genetic material and, more strikingly, the loss of as much as 13% of gene functions [38]. Whether selection for synthesis of hypoacylated LPS occurred during transition to the flea vector or within the mammalian host is unknown. Our humanized mouse model should help shed light on the molecular mechanisms involved in the selection of microbe as well as host, and offer a platform in which to readily test possible prophylactic and therapeutic interventions.

## Materials and Methods

### Ethics statement

This study was carried out in strict accordance with the recommendations in the Guide for the Care and Use of Laboratory Animals of the National Institutes of Health. All protocols were approved by the Institutional Animal Care and Use Committee of the University of Washington.

### Reagents

Ultrapure O111:B4 EC LPS was purchased from InvivoGen (San Diego, CA), synthetic lipid IVa from Peptides International Inc. (Louisville, KY), and ODN1826 from Coley Pharmaceuticals (prior to acquisition by Pfizer, Düsseldorf, Germany). Antibodies for flow cytometry were from Becton Dickinson (Franklin Lakes, NJ), eBioscience (San Diego, CA), or BioLegend (San Diego, CA).

### LPS purification and analysis

LPS was purified by Mg^2+^-ethanol precipitation as described by Darveau and Hancock [39] for *P. aeruginosa* and hot water-phenol extraction [40] for *Y. pestis*. LPS was further purified by Folch extraction [41] and phenol extraction [42] and resuspended in endotoxin-free water. For mass spectrometric analysis, lipid A was isolated by hydrolysis of LPS in 1% SDS at pH 4.5 [43]. Negative-ion matrix-assisted laser desorption ionization–time of flight (MALDI-TOF) mass spectrometry was performed as described previously [44], [45]. Lyophilized lipid A was dissolved with 10 µl of 5-chloro-2-mercaptobenzothiazole (Sigma-Aldrich, St. Louis, MO) MALDI matrix in chloroform-methanol at 1∶1 (vol/vol) and applied (1 µl) to the sample plate. Experiments were performed using a Bruker Autoflex III MALDI-TOF mass spectrometer (Bruker Daltonics, Inc., Billerica, MA). Each spectrum was an average of 300 shots. External calibration was performed with ES Tuning Mix (Agilent, Palo Alto, CA). Similar results were observed with multiple independent LPS preparations from YP and PA.

### Luciferase assay for TLR4 stimulations

HEK-293 cells were transiently transfected in 96-well flat-bottom plates as described [16], with the following amounts of DNA per well: 10 ng ELAM-Luc, 0.2 ng *Renilla*-Luc, 0.0025 µg hu or mu CD14, 0.0005 µg muTLR4, 0.002 µg huTLR4, 0.005 µg muMD-2, 2.5 ng huMD-2. Total DNA per well was normalized to 50 ng by the addition of empty vector. Cells were stimulated as described in the presence of 10% heat-inactivated serum.

### Mice

Human genomic BAC RP11-150L1 (*TLR4*) and RP11-592J7 (*MD-2*), obtained from The BACPAC Resource Center (CHORI, Oakland, CA), were injected into fertilized B6C3×B6 oocytes by the Transgenic Core at the University of Washington. We chose BACs that contained at least 50 Kb of human genomic sequences flanking *TLR4* and *MD-2* to direct tissue-specific expression in the mice. The resulting founders were backcrossed initially to *TLR4* KO ([46] provided by S. Akira) and *MD-2* KO ([47] provided by D. Golenbock) respectively and later to double-KO mice, on a C57BL/6J background, with genotypes assessed by PCR. Illumina SNP panels were used to verify backcross level and generate congenics more efficiently (Illumina, Inc., San Diego, CA). At backcross 9 (NE9), a sampling of humanized mice matched C57BL/6 SNPs at 350–352 of 377 loci (93%). Mice labeled as KO in the figures are mainly littermate *MD2* BAC+ but *TLR4* BAC- on a DKO background although we also included DKO or single KO mice. We confirmed by PCR and sequencing that all the mice used for the *S.* Typhimurium infections were *Nramp1^D169^*, the sensitive allele found in WT B6 mice [48]. Mice ranged in age between 7 and 26 weeks.

### Transgenic BAC copy number

Copy number of the BAC transgene was determined by real-time PCR using an ABI 7300 Real Time PCR System and the Brilliant II SYBR Green QPCR reagents from Stratagene (now Agilent Technologies Inc., Santa Clara, CA). A primer pair that can amplify a 59-bp product from either mouse or human *TLR4* sequence was designed using Primer Express as follows: *TLR4*, forward, 5′-CTCTGCCTTCACTACAGAGAC-3′; reverse, 5′-TGGATGATGTTGGCAGCAATG-3′. For hu*MD-2*, the forward primer was 5′-GTTACTGATCCTCTTTGCATTTG-3′ and the reverse primer 5′-CTGCTTCTGAGCTTCAGTAAATATG-3′, that amplified a 109-bp product. For mu*MD-2*, the forward primer was 5′-GTCCGATGGTCTTCCTGGCGAGT-3′ and the reverse primer 5′-GCTTCTCAGATTCAGTCAATATGGG-3′, that amplified a 108-bp product. *TBP* (TATA-binding protein) gene was amplified as an endogenous control to account for variability in the concentration of the genomic DNA used in the PCR reaction [22]. Genomic DNA extracted from a heterozygous mouse was used as the calibrator (i.e., copy number = 1) in the Pfaffl method [49] to calculate the relative copies of the transgene. Experimentally, serial dilutions of genomic DNA extracted from several transgenic mice were added to the real-time PCR mix according to manufacturer recommendations. The amplification was performed with an initial 2 min incubation at 50°C and 10 min at 95°C, followed by 40 cycles of 95°C, 15 s and 60°C, 1 min, and then specificity of amplification was assessed using a melt-curve analysis. The hu*MD-2* transgenic BAC line has 2 copies of the transgene, whereas one line of the hu*TLR4* transgenic BAC mice has 2 copies and a second line has 4 copies.

### Expression levels

Relative TLR4 expression levels were determined using real-time PCR and the Brilliant II SYBR Green QPCR reagents. Oligo dT primed cDNA was reverse transcribed from whole RNA isolated from various organs using Superscript II (Invitrogen, Carlsbad, CA). Following DNase treatment, amplification was performed as described for copy number (above) and ß -actin was used to normalize the data between samples. The ß -actin forward primer was 5′-TCCTTCGTTGCCGGTCCAC-3′ and the reverse primer 5′-ACCAGCGCAGCGATATCGTC-3′. Minus-RT controls were run for each sample.

### Bone-marrow-derived macrophages

BMDM were generated by culturing BM cells in RPMI+glutamax+10% HI-FCS+Pen/Strep+15% L929 conditioned medium (LCM) for a total of 8–9 days. ∼4×10^7^ BM cells were plated in 15 cm uncoated dishes in 25 ml medium. 20 ml of fresh medium was added 3 days later. Cells were harvested in PBS+3 mM EDTA, spun down, and resuspended in medium without LCM. 10^6^ cells were plated per well in 96-well flat bottom plates and supernatants were harvested after 4 hr of stimulation. Cytokines were measured by Luminex. At the time of harvest, cells were stained with F4/80 FITC, CD11b PerCP Cy5.5, and either PE-labeled anti-mouse TLR4 (clone MTS510) or its isotype control (rat IgG2a), or with anti-human TLR4 (clone HTA125) or its isotype control (muIgG2a).

### Mouse infections

*S.* Typhimurium strain SL1344 was grown overnight in LB, pelleted, resuspended in PBS, and diluted to an appropriate concentration based on the O.D. at 600 nm. The inoculum was administered in 200 µl PBS intraperitoneally; actual CFU administered was verified by plating on LB plates (1,720 and 1,680 CFU, respectively). For *Y. pestis*, a frozen aliquot of strain CO92 that had been grown at 37°C was thawed at the time of infection and serially diluted in PBS to achieve appropriate inoculum. 100 µl were injected subcutaneously between the shoulder blades of mice anesthetized with isoflurane. Actual CFU administered were verified by plating on blood-agar plates (184 and 227 CFU, respectively). Mice were monitored twice daily, blinded to genotype, beginning day 3 post-infection using a humane endpoint scoring system. For *Y. pestis* CFU determination, actual CFU administered per experiment were 254, 344, 253, 257, and 262. Tissues were homogenized in PBS using disposable gentleMACS C Tubes (Miltenyi Biotec, Bergisch Gladbach, Germany) and the Omni MultiMix 200 homogenizer (Omni International, Kennesaw, GA). Two mice were moribund (one WT and one hu-2 copy) at the time of harvest and were not included in the analysis. For Fig. S5, a frozen aliquot of *Y. pestis* CO92 grown at 26°C was used.

### Whole spleen flow cytometry assay

Spleens were mechanically dissociated between glass slides and RBC-lysed single cell suspensions were stimulated in round-bottom 96-well plates in the presence of 10 µg/ml Brefeldin-A for 4 hr. Wells were treated with 2 mM EDTA for 10 minutes to detach any cells that might have adhered during stimulation. Cells were stained, following blockade of Fc receptors, with CD11b PerCP-Cy5.5, CD11c PE-Cy7, CD19 APC-Cy7, and CD3 APC. Cells were then lysed with cytofix/cytoperm solution (Becton Dickinson) and stained for intracellular TNF (FITC) and IL-12/IL-23 p40 (PE). 500,000 to 1 million events were collected on a BD FACSCanto and results were analyzed using FlowJo (Tree Star, Inc. Ashland, OR).

### Luminex

Serum samples or BMDM culture supernatants were filtered in a 96-well 1.2 µm membrane HTS plate (Millipore, Billerica, MA) and diluted 1∶3 or 1∶5 in serum diluent, or 1∶3 in culture medium, respectively. Cytokines and chemokines (IL-1ß, IL-6, IL-10, IL-12p40, IL-12p70, TNFa, CCL5) were quantified using a Bio-Plex 200 system with Bio-Plex Pro Assay Kits (Bio-Rad, Hercules, CA) following the manufacturer's instructions.

### Statistics

Prism software (GraphPad, La Jolla, CA) was used for all statistical analyses with test indicated in the figure legend or table.

## Supporting Information

Figure S1 **Structural diversity of lipid A in Gram-negative microorganisms.** Chemical structures of (A) hexa-acylated *Escherichia coli* grown at 37°C, (B) hexa-acylated *Yersinia pestis* grown at 26°C, (C) tetra-acylated *Yersinia pestis* grown at 37°C, (D) penta-acylated *Yersinia pestis* grown at 37°C, (E) penta-acylated *Pseudomonas aeruginosa* grown at 37°C, and (F) tetra-acylated Lipid IVa, a precursor in lipid A biosynthesis.
(TIF)

Figure S2 **TLR4 RNA expression in various tissues from humanized mice compared to WT mice.** Real-time PCR was performed on total RNA extracted from indicated tissues and normalized to ß-actin levels. Expression in WT tissues was set at 1 and fold change in 4- and 2-copy humanized and KO mice are shown (mean+/−SEM of 3 repeats). SM, skeletal muscle.
(TIF)

Figure S3 **Normal development of splenic subsets identified in** **Fig. 4A** **in humanized mice.** Shown are the % of cells in each gate from all the mice analyzed by flow cytometry (N = 9 WT, 8 4-copy and 5 2-copy humanized, and 9 KO mice). The line represents the median and data were analyzed using 1-way ANOVA followed by Dunn's post-test for multiple pairwise comparisons. Significant differences are shown. * *P*<0.05.
(TIF)

Figure S4 **Macrophages/monocytes express human TLR4 at similar levels in the 2-copy and 4-copy humanized mice.** (A) Splenocytes from WT, 2-copy and 4-copy humanized TLR4/MD-2, and KO mice were stained for surface markers as in Fig. 4A to identify the various cell populations listed. B-cells were identified as CD19+ cells and T-cells as CD3+ cells. In addition, cells were stained with either PE-labeled anti-mouse TLR4 (blue histograms), or with anti-human TLR4 (red histogram), or with isotype controls (filled grey histograms).
(TIF)

Figure S5 **Recognition of** ***Y. pestis*** **inoculum by TLR4 does not affect increased susceptibility of humanized TLR4/MD-2 mice.** Mice were infected with 100 CFU hexa-acylated *Y. pestis* subcutaneously. Mice were monitored twice daily, blinded to genotype, beginning day 3 post-infection using a humane endpoint scoring system. N = 6 per genotype. The log-rank test was used to compare the survival curves. **P<0.01, * *P*<0.05.
(TIF)

Table S1 **Two-Way ANOVA with Bonferroni posttests of HEK-293 data from Fig. 1.**
(DOC)

Table S2 **Two-Way ANOVA with Bonferroni posttests of mac/mono TNF and cDC IL-12/23 p40 response from Fig. 4.**
(DOC)
