# Supplementary figures and images for: Humanized TLR4/MD-2 Mice Reveal LPS Recognition Differentially Impacts Susceptibility to Yersinia pestis and Salmonella enterica
Source: PLoS Pathog. 2012 Oct 11;8(10):e1002963. doi: 10.1371/journal.ppat.1002963 (PMC3469661; doi:10.1371/journal.ppat.1002963)

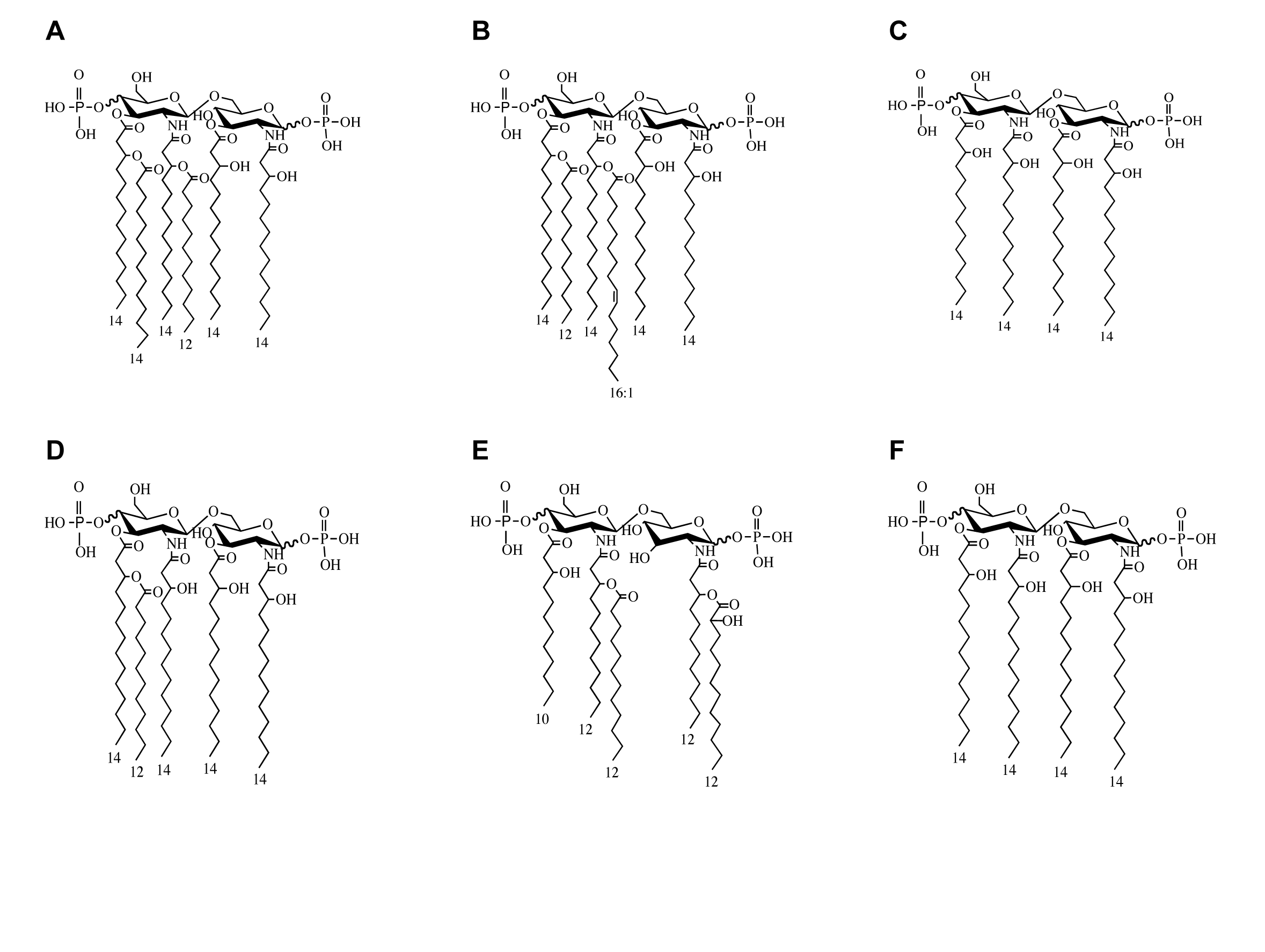

Supplement: Figure S1 — Structural diversity of lipid A in Gram-negative microorganisms. Chemical structures of (A) hexa-acylated Escherichia coli grown at 37°C, (B) hexa-acylated Yersinia pestis grown at 26°C, (C) tetra-acylated Yersinia pestis grown at 37°C, (D) penta-acylated Yersinia pestis grown at 37°C, (E) penta-acylated Pseudomonas aeruginosa grown at 37°C, and (F) tetra-acylated Lipid IVa, a precursor in lipid A biosynthesis. (TIF) [file ppat.1002963.s001.tif]

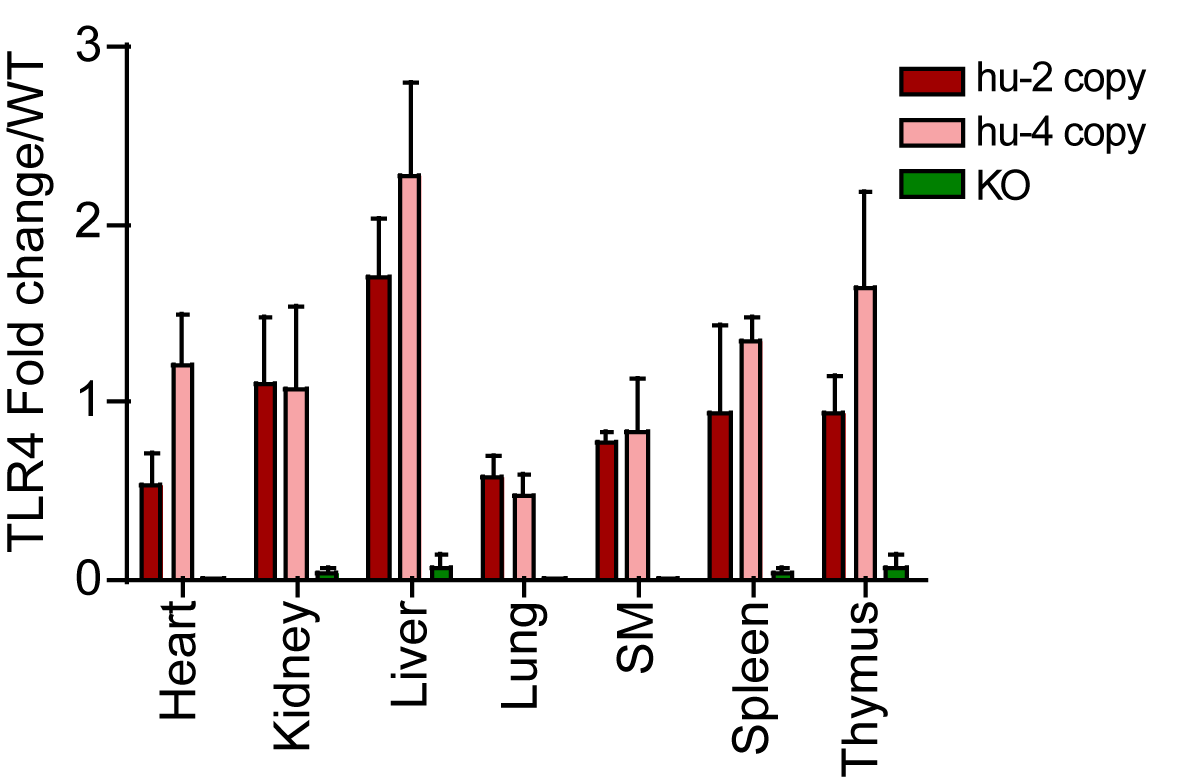

Supplement: Figure S2 — TLR4 RNA expression in various tissues from humanized mice compared to WT mice. Real-time PCR was performed on total RNA extracted from indicated tissues and normalized to ß-actin levels. Expression in WT tissues was set at 1 and fold change in 4- and 2-copy humanized and KO mice are shown (mean+/−SEM of 3 repeats). SM, skeletal muscle. (TIF) [file ppat.1002963.s002.tif]

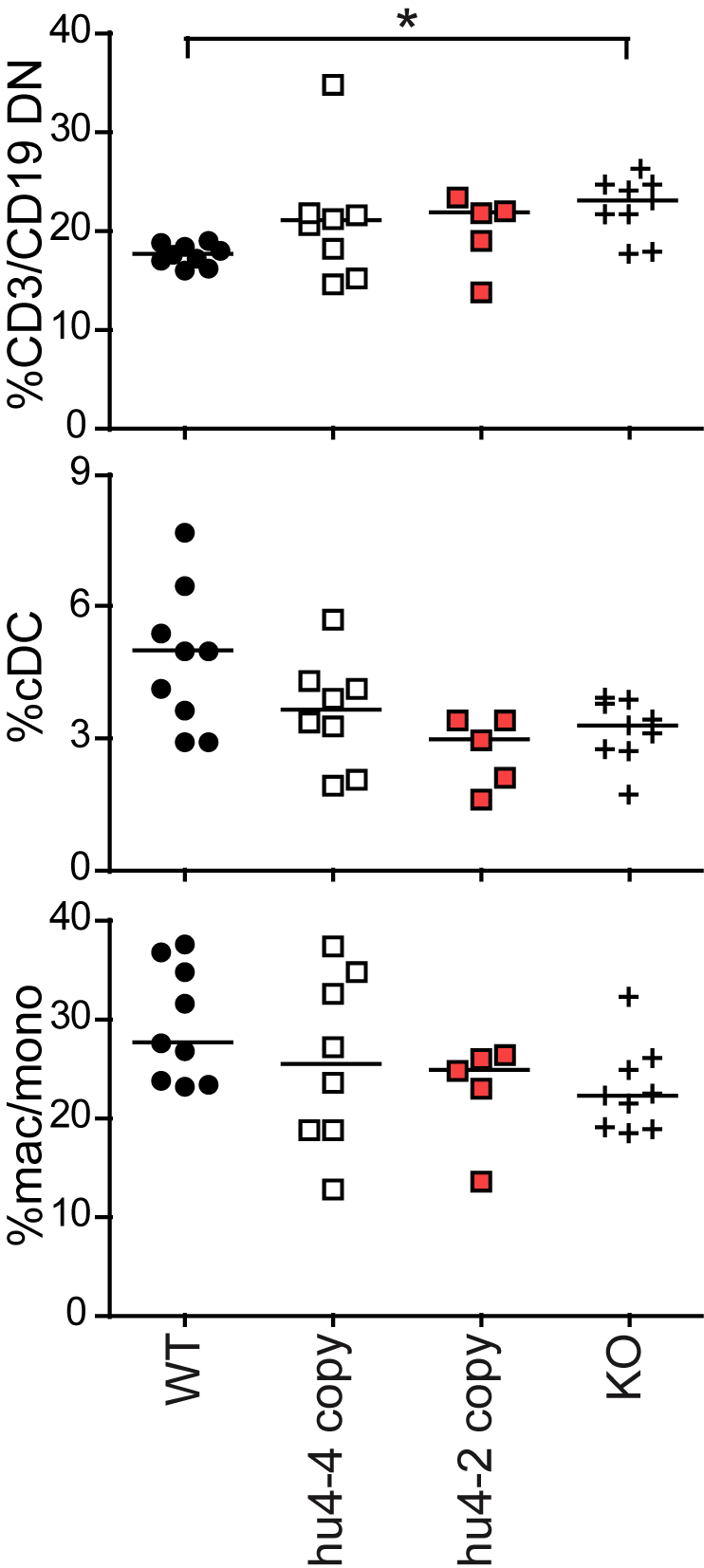

Supplement: Figure S3 — Normal development of splenic subsets identified in Fig. 4A in humanized mice. Shown are the % of cells in each gate from all the mice analyzed by flow cytometry (N = 9 WT, 8 4-copy and 5 2-copy humanized, and 9 KO mice). The line represents the median and data were analyzed using 1-way ANOVA followed by Dunn's post-test for multiple pairwise comparisons. Significant differences are shown. * P<0.05. (TIF) [file ppat.1002963.s003.tif]

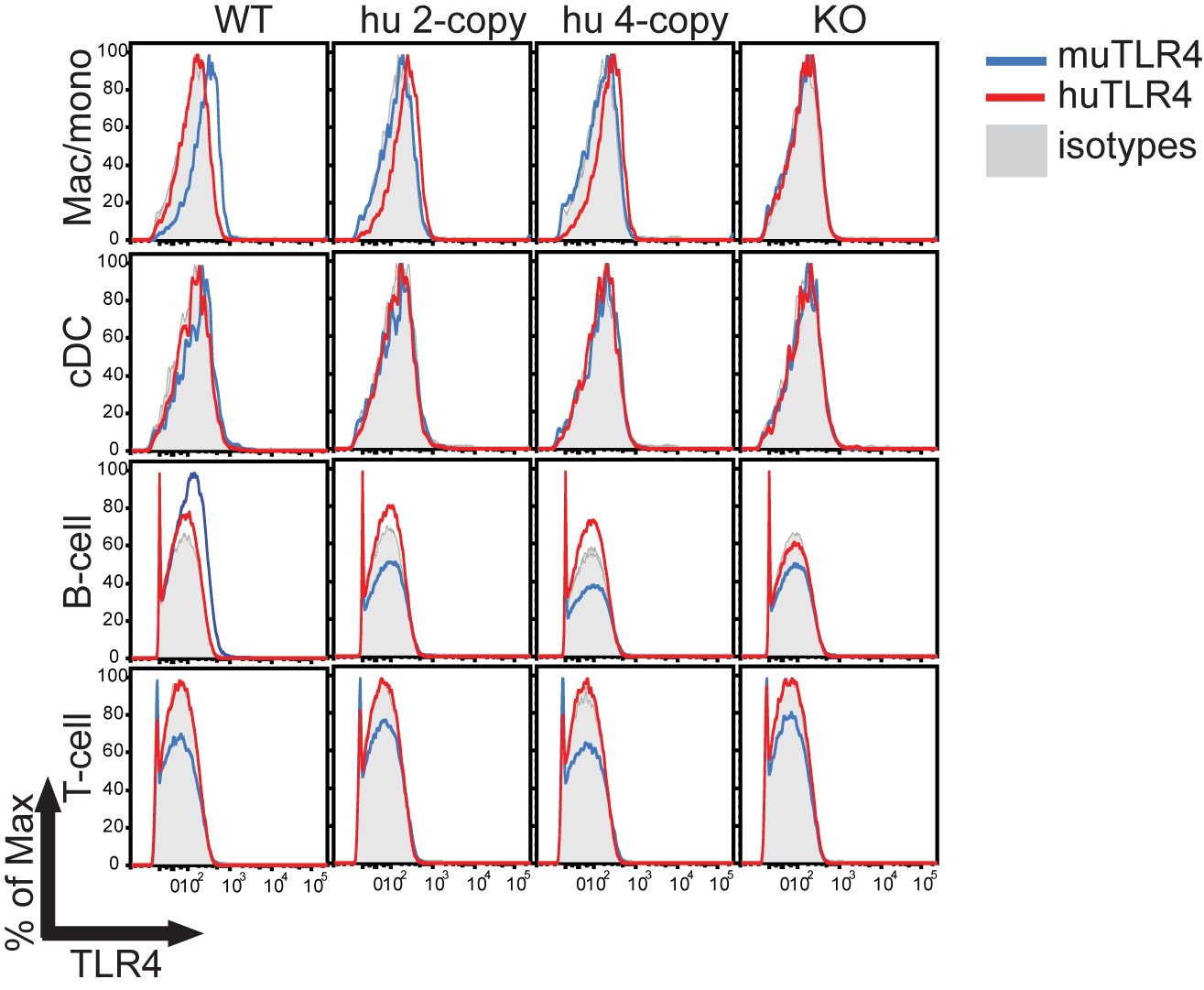

Supplement: Figure S4 — Macrophages/monocytes express human TLR4 at similar levels in the 2-copy and 4-copy humanized mice. (A) Splenocytes from WT, 2-copy and 4-copy humanized TLR4/MD-2, and KO mice were stained for surface markers as in Fig. 4A to identify the various cell populations listed. B-cells were identified as CD19+ cells and T-cells as CD3+ cells. In addition, cells were stained with either PE-labeled anti-mouse TLR4 (blue histograms), or with anti-human TLR4 (red histogram), or with isotype controls (filled grey histograms). (TIF) [file ppat.1002963.s004.tif]

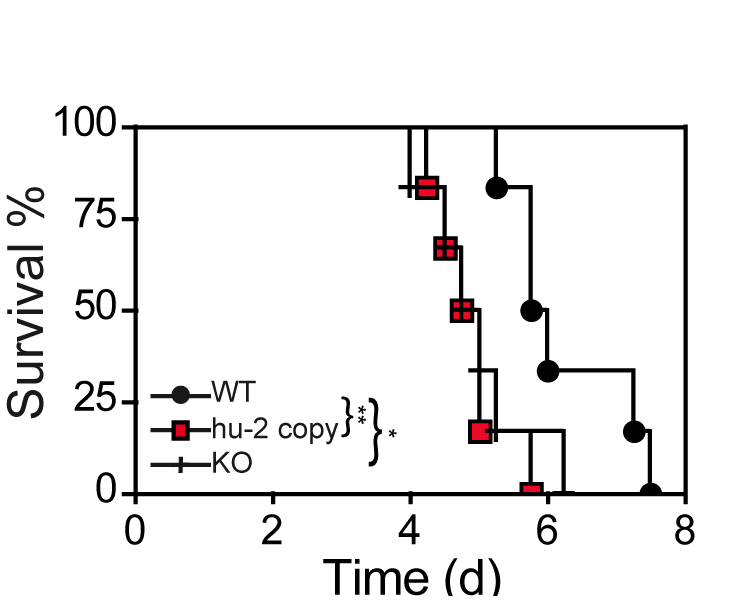

Supplement: Figure S5 — Recognition of Y. pestis inoculum by TLR4 does not affect increased susceptibility of humanized TLR4/MD-2 mice. Mice were infected with 100 CFU hexa-acylated Y. pestis subcutaneously. Mice were monitored twice daily, blinded to genotype, beginning day 3 post-infection using a humane endpoint scoring system. N = 6 per genotype. The log-rank test was used to compare the survival curves. **P<0.01, * P<0.05. (TIF) [file ppat.1002963.s005.tif]
