# Supplementary material for: Humanized TLR4/MD-2 Mice Reveal LPS Recognition Differentially Impacts Susceptibility to Yersinia pestis and Salmonella enterica
Source: PLoS Pathog. 2012 Oct 11;8(10):e1002963. doi: 10.1371/journal.ppat.1002963 (PMC3469661; doi:10.1371/journal.ppat.1002963)
Supplement: Table S1 — Two-Way ANOVA with Bonferroni posttests of HEK-293 data from Fig. 1. (DOC) [file ppat.1002963.s006.doc]

| ng/ml | muTLR4  muMD-2  muCD14 | | | huTLR4  huMD-2  huCD14 | | | muTLR4  muMD-2  huCD14 | | | huTLR4  huMD-2  muCD14 | | | muTLR4  muMD-2  no CD14 | | | huTLR4  huMD-2  no CD14 | | |
| --- | --- | --- | --- | --- | --- | --- | --- | --- | --- | --- | --- | --- | --- | --- | --- | --- | --- | --- |
| **1000** | PA | YP | L-IVa | PA | YP | L-IVa | PA | YP | L-IVa | PA | YP | L-IVa | PA | YP | L-IVa | PA | YP | L-IVa |
| EC | n.s. | ** | *** | *** | *** | *** | n.s. | *** | n.s. | *** | *** | *** | *** | *** | *** | *** | *** | *** |
| PA |  | n.s. | *** |  | ** | *** |  | n.s. | *** |  | * | *** |  | *** | n.s. |  | ** | *** |
| YP |  |  | *** |  |  | *** |  |  | * |  |  | *** |  |  | *** |  |  | *** |
|  |  |  |  |  |  |  |  |  |  |  |  |  |  |  |  |  |  |  |
| **100** | PA | YP | L-IVa | PA | YP | L-IVa | PA | YP | L-IVa | PA | YP | L-IVa | PA | YP | L-IVa | PA | YP | L-IVa |
| EC | n.s. | *** | *** | *** | *** | *** | n.s. | *** | n.s. | *** | *** | *** | n.s. | *** | * | *** | *** | *** |
| PA |  | n.s. | *** |  | * | *** |  | n.s. | *** |  | *** | *** |  | *** | n.s. |  | n.s. | *** |
| YP |  |  | n.s. |  |  | *** |  |  | * |  |  | *** |  |  | *** |  |  | *** |
|  |  |  |  |  |  |  |  |  |  |  |  |  |  |  |  |  |  |  |
| **10** | PA | YP | L-IVa | PA | YP | L-IVa | PA | YP | L-IVa | PA | YP | L-IVa | PA | YP | L-IVa | PA | YP | L-IVa |
| EC | n.s. | *** | *** | *** | *** | *** | n.s. | *** | *** | *** | *** | *** | *** | *** | *** | *** | *** | *** |
| PA |  | *** | *** |  | n.s. | *** |  | *** | *** |  | ** | *** |  | *** | n.s. |  | n.s. | *** |
| YP |  |  | * |  |  | *** |  |  | n.s. |  |  | *** |  |  | *** |  |  | n.s. |
|  |  |  |  |  |  |  |  |  |  |  |  |  |  |  |  |  |  |  |
| **1** | PA | YP | L-IVa | PA | YP | L-IVa | PA | YP | L-IVa | PA | YP | L-IVa | PA | YP | L-IVa | PA | YP | L-IVa |
| EC | *** | n.s. | *** | *** | n.s. | n.s. | *** | n.s. | *** | *** | n.s. | n.s. | n.s. | n.s. | *** | n.s. | n.s. | n.s. |
| PA |  | *** | *** |  | *** | *** |  | *** | *** |  | *** | *** |  | n.s. | ** |  | n.s. | n.s. |
| YP |  |  | *** |  |  | n.s. |  |  | *** |  |  | n.s. |  |  | *** |  |  | n.s. |

L-IVa: Lipid IVa

**P*<0.05, ***P*<0.01, ****P*<0.001, n.s. not significant
